# Application of Polymeric CO_2_ Thickener Polymer-Viscosity-Enhance in Extraction of Low-Permeability Tight Sandstone

**DOI:** 10.3390/polym16020299

**Published:** 2024-01-22

**Authors:** Hong Fu, Kaoping Song, Yiqi Pan, Hanxuan Song, Senyao Meng, Mingxi Liu, Runfei Bao, Hongda Hao, Longxin Wang, Xindong Fu

**Affiliations:** 1 Unconventional Oil and Gas Research Institute, China University of Petroleum, Beijing 102249, China; 2 College of New Energy and Materials, China University of Petroleum, Beijing 102249, China; 3 School of Petroleum and Natural Gas Engineering, School of Energy, Changzhou University, Changzhou 213164, China; 4 Daqing Drilling Engineering Company Underground Operation Company Technology Research Institute, Songyuan 138000, China

**Keywords:** low-permeability sandstone, CO_2_ flooding, polymeric thickener, MMP reduction, molecular simulation

## Abstract

The conventional production technique employed for low-permeability tight reservoirs exhibits limited productivity. To solve the problem, an acetate-type supercritical carbon dioxide (scCO_2_) thickener, PVE, which contains a large number of microporous structures, was prepared using the atom transfer radical polymerization (ATRP) method. The product exhibited an ability to decrease the minimum miscibility pressure of scCO_2_ during a solubility test and demonstrated a favorable extraction efficiency in a low-permeability tight core displacement test. At 15 MPa and 70 °C, PVE-scCO_2_ at a concentration of 0.2% exhibits effective oil recovery rates of 5.61% for the 0.25 mD core and 2.65% for the 5 mD core. The result demonstrates that the incorporation of the thickener PVE can effectively mitigate gas channeling, further improve oil displacement efficiency, and inflict minimal damage to crude oil. The mechanism of thickening was analyzed through molecular simulation. The calculated trend of thickening exhibited excellent agreement with the experimental measurement rule. The simulation results demonstrate that the contact area between the polymer and CO_2_ increases in direct proportion to both the number of thickener molecules and the viscosity of the system. The study presents an effective strategy for mitigating gas channeling during scCO_2_ flooding and has a wide application prospect.

## 1. Introduction

The development of unconventional oil and gas fields is increasingly reliant on tight oil reservoirs, which had a global technically recoverable reserve of approximately 480.28 × 10^8^ t in 2021, accounting for merely 4.96% of the total global reserves [1]. It is important to note that there still exists significant untapped potential in the field of tight oil development. A low-permeability tight reservoir exhibits characteristics of tight lithology, low porosity and permeability, prominent non-Darcy flow behavior, low pressure coefficient, amplitude traps, and low natural productivity. The tight reservoirs in China, predominantly lacustrine in nature, exhibit a lower reservoir pressure coefficient, significant heterogeneity [2], and relatively low maturity when compared to the marine tight oil formations found in North America [3]. The initial development phase in China included natural depletion mining and water flooding mining, resulting in a significant deficiency in formation energy and waterflooding causing heavy-scale precipitation [4]. The current approach to enhanced oil recovery primarily involves the combination of horizontal well fracturing, water flooding enhancement, and various auxiliary techniques [5,6]. The utilization of gas flooding has gradually gained global recognition among scientists due to its abundant sources, diverse composition, and effective maintenance of formation pressure. The adaptation range varies among gases, with specific advantages and disadvantages outlined in Table 1.

Carbon dioxide (CO_2_) flooding technology has garnered significant attention in the realm of low-permeability tight reservoirs due to its dual benefits of recovery and emission reduction [12]. Experimental findings from the Bakken and Eagle Ford formations, which exhibit notable recovery effects, demonstrate that the injection of carbon dioxide effectively penetrates heterogeneous rock, facilitating the enhanced recovery of residual oil within the matrix [13,14]. A physical simulation test of oil displacement in a Daqing block has also confirmed that carbon dioxide flooding can enhance the recovery of crude oil with large and medium pores in low-permeability cores [15]. The continuous increase in temperature and pressure beneath the reservoir results in the transformation of CO_2_ into a supercritical state, which enhances its solubility in crude oil. Additionally, the higher compression ratio contributes to an expansion in the volume of crude oil. The significant disparity in viscosity between crude oil and CO_2_, however, gives rise to challenges such as flow disturbances and environmental acidification [16]. Scientists have put forward two types of solutions for this purpose: The first approach is to decrease the viscosity of crude oil by introducing light hydrocarbons or microorganisms. The alternative is to enhance the viscosity of the displacement phase by incorporating suitable additives into carbon dioxide to form a carbon dioxide foam or a uniform thickening system [17,18,19]. Taking into account the current state of technological development, as well as considerations for production safety and economic factors, introducing a thickening agent into supercritical carbon dioxide (scCO_2_) to mitigate the viscosity difference between the two phases will overcome the aforementioned technical bottleneck [20], enhance displacement phase performance, and facilitate residual oil recovery.

Reputable scholars, both domestic and international, have conducted extensive research on the development and application of carbon dioxide thickeners. The current understanding is that the most effective thickeners contain fluorine and silicon [21,22]. However, the cost of silicon-based thickeners is high, and the presence of toxic or harmful components such as fluorine and ether in some thickeners causes significant damage to water and soil and cannot meet the current requirements of green development [23,24,25]. Acrylamide thickeners exhibit sensitivity to injection pressure, and the incorporation of fibers/microspheres yields favorable results, albeit at a higher cost [26,27,28]. The development of cost-effective and environmentally friendly thickeners is therefore imperative. The ideal thickener for oil displacement should solely rely on the functional groups composed of C, H, and O in the molecule to achieve its purpose without introducing any hydrocarbons or other elements that are not present in CO_2_. The ideal thickener should minimize the addition of cosolvent while demonstrating excellent adaptability to the reservoir environment. The thickening mechanism of an acetate thickener is analogous to that of a hydrocarbon-based thickener [29], and its structure also fulfills the requirement for an ideal thickener by not introducing any additional elements. Polyvinyl acetate ester exhibits excellent lipophilicity, eliminating the need for any additional additives during its application process and facilitating cost control. However, the utilization of polyvinyl acetate ester (PVAC) in its pure form often leads to a molecular volume that is too large to permeate through the porous medium effectively, resulting in limited thickening efficacy that fails to meet the requirements of practical applications.

The present study involves the synthesis of a type of environmentally friendly thickener (Polymer-Viscosity-Enhance, PVE) that can adapt to the requirements of CO_2_ flooding in tight sandstone reservoirs. In this paper, the solubility, thickening performance, and thickener displacement effect are evaluated for PVE, and the influence of the displacement agent on the core and crude oil composition is investigated. In addition, the team analyzed the thickening mechanism from the perspective of the microscopic mechanism, providing theoretical support for the field application of thickener/scCO_2_ flooding.

## 2. Materials and Methods

### 2.1. SEM Measurement of the Rock Samples

A low-permeability tight sandstone core (Φ38 mm * 200 mm) from a block in Daqing Oilfield was selected, with gas permeability measurements recorded as 0.25 mD and 5 mD. The fresh sandstone surface was subjected to gold spraying scanning using a scanning electron microscope, enabling the observation of microstructure distribution in the core section.

### 2.2. Method for Producing Thickening Agent—PVE

**Figure 1:**
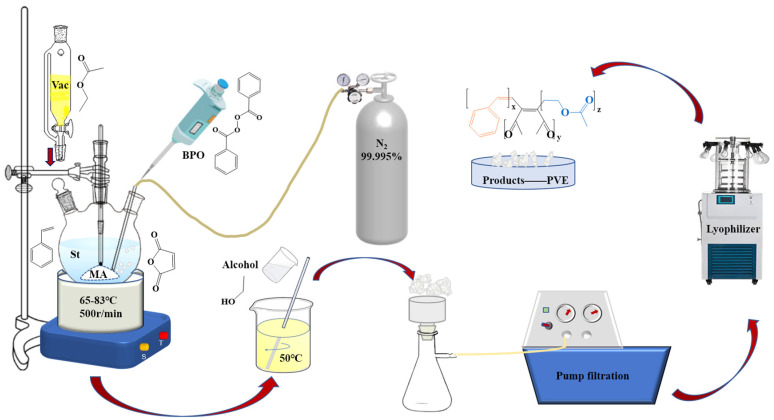
The diagram of the PVE synthesis device.

An MA-ST aqueous solution was prepared in a deoxygenated environment by combining maleic anhydride (MA, Shanghai Aladdin Biochemical Technology Co., Ltd., Shanghai, China) and styrene (St, Shanghai Aladdin Biochemical Technology Co., Ltd.) in a molar ratio of 4:1. Subsequently, the solution was transferred into a 500 mL four-port flask. The solution was heated in a water bath with continuous stirring. When the temperature exceeded 70 °C, the initiator benzoyl peroxide (BPO, Shanghai Aladdin Biochemical Technology Co., Ltd.) was introduced, followed by the addition of vinyl acetate (VAC, Shanghai Aladdin Biochemical Technology Co., Ltd.) through a separation funnel. The control VAC was dripped at a flow rate for one hour, followed by a constant-temperature reaction for two hours before the reaction was stopped and the temperature was turned off. After being cooled to 50 °C, the light-yellow viscous liquid in the bottle was directly mixed with anhydrous ethanol absolute (99.5%, Sinopharm Group Chemical Reagent Co., Ltd., Beijing, China) for solvent water and incomplete monomer removal. After the solid was extracted and filtered, the crude product was transferred to a freeze-dryer (FD-1D-110, BIOCOOL (Beijing) Instrument Co., Ltd., Beijing, China) for a duration of 2 h. Subsequently, the final product, PVE, was obtained in loose white powder form, and it underwent grinding into 100~200 mesh particles for further utilization. The synthesis flow chart is shown in Figure 1.

### 2.3. FT-IR Measurement of PVE

The appropriate quantity of product powder was purified and subsequently compressed using potassium bromide. Following confirmation of the completion of compression, the synthesized product was characterized utilizing the Spectrum Two L1600401 infrared spectrometer (PerkinElmer, Waltham, MA, USA), and the infrared absorption spectrum of the product was obtained.

### 2.4. NMR Measurement of PVE

The structure of the synthesized polymer was characterized using a Bruker-400 MHz NMR instrument (Brucker, Beijing, China) with ^13^C NMR spectroscopy capability; tetramethylsilane (TMS) served as the internal standard, and D_2_O was employed as the solvent.

### 2.5. SEM Measurement of PVE

Conductive adhesive was reapplied onto the sample table, and gold (platinum) spraying was set to a constant speed for 10 min.

### 2.6. TG and DSC Measurement of PVE

The temperature–mass correlation of the sample was determined utilizing a differential thermogravimetric synchronous analyzer, which regulated the temperature within an oxygen atmosphere from 0 to 800 °C at a heating rate of 5 °C/min. Ultimately, the heat loss spectrum was acquired. The thermal stability of the product was assessed using the heat loss curve and its corresponding differential curve (the testing protocol followed ISO 11358-1:222 as a reference standard) [30].

### 2.7. Solubility Test of PVE in scCO_2_

The thickening agent was added to a 350 mL high-temperature and high-pressure variable-speed visual reactor (temperature measuring range, 20~180 °C; pressure, 40 MPa; homemade) at a mass fraction of 0.1%, and enough CO_2_ was injected into the reactor at room temperature. The system temperature was set to 70 °C, and the pressure of the system was raised to 7.84 MPa or higher using a pressurizing device. To ensure complete dissolution of the polymer, the PVE-scCO_2_ mixture was stirred at a low speed (300 r/min) for 5 min and then allowed to stand under constant pressure for an additional 5 min. The system state in the reactor was continuously monitored during the pressurization process, and the feedback number of the pressure probe was recorded in the window when the system underwent phase change (minimum miscible pressure). To ensure experimental accuracy, the autoclave system pressure was adjusted by manipulating the hand pump near the critical point of phase transformation to obtain an average value. A control group without any treatment was established, and pure CO_2_ was injected into the system to maintain consistent environmental conditions with those of the thickener-treated system.

### 2.8. Evaluation of Viscosification Effect of PVE

The fully dissolved PVE-scCO_2_ mixture was introduced into a closed Harker Mars 60 rheometer (Thermo Fischel, Dreieich, Germany), which was designed to withstand high pressure, acid corrosion, and temperature fluctuations at 70 °C. The Job Manager module in the Haake RheoWin software 4 was selected for automatic process control of measurement and analysis procedures. The system was pre-sheared for 10 min to ensure the thorough mixing of PVE and scCO_2_, with the shear rate set at 1000 s^−1^. After pre-shear, the shear rate was adjusted to 170 s^−1^, and the system was incrementally heated at 10, 15, and 20 MPa to investigate the variation in the shear viscosity of the system. The control group was composed solely of pure scCO_2_.

### 2.9. Assessment of Oil Displacement Effect

The core holder (Φ38 mm × 30 mm) was placed in a 70 °C incubator. The mixture, which was fully mixed with 0.2% PVE and scCO_2_, was achieved by stirring with a piston, followed by displacement until the completion of the experiment. The natural core was subjected to scCO_2_ flooding at an injection pressure of 15 MPa to simulate the formation environment. The displacement rate was measured at 0.02 mL/min. The investigation focused on the displacement efficiency of cores with varying permeability and the impact of introducing PVE.

### 2.10. Gas–Oil Relative Permeability Measurement

The experiment reference was ASTM D4525-13-Standard [31] Test Method for Permeability of Rocks by Flowing Air. A natural outcrop core was measured using dry air as the tested medium, and the gas phase permeability curve trend of different cores was compared before and after 0.2% PVE displacement. The oil phase permeability Kro was determined with reference to MNL7320140019 “Reservoir Types and Characterization” [32].

### 2.11. Analysis of the Fluid Produced

The oil samples before and after displacement were collected, dehydrated, and subjected to analysis using an Agilent 7890 A (Santa Clara, CA, USA) gas chromatograph. The test was conducted using column chromatography. Normal hexane was employed for the precipitation and filtration of an asphaltene solution in rock soluble organic matter or crude oil. Subsequently, the filtrate was subjected to washing with organic solvents of varying polarity, resulting in the sequential acquisition of saturated hydrocarbon, aromatic hydrocarbon, and colloid solutions. Finally, the volatile solvent from the component solution was weighed until a constant weight was achieved to determine the mass fraction of components present in the sample.

### 2.12. Molecular Dynamics (MD) Simulation of Thickening Mechanism

The thickening mechanism of the system was analyzed through a classical molecular simulation, based on the observed phenomenon that carbon dioxide effectively enhances production when it undergoes thickening [33]. GROMACS-2020.6 software and the charmm36 force field were employed for conducting molecular dynamics simulations [34,35]. The molecular dynamics (MD) simulation was conducted with constant pressure, temperature, and three-dimensional periodic boundary conditions (PBCs). The Berendsen, V-rescale, and Nose–Hoover thermostats were employed for temperature control in the NVT ensemble equilibration, NPT ensemble equilibration, and final product simulation [36,37]. The changes in viscosity and contact area of the system were determined based on the final equilibrium structure under different concentrations and environmental conditions [38]. To optimize energy reduction, an initial uniform distribution was set for the structure, and a blank control for viscosity was implemented in the pure CO_2_ system with a specific number of molecules.

## 3. Results and Discussion

### 3.1. Rock Micromorphology Analysis

**Figure 2:**
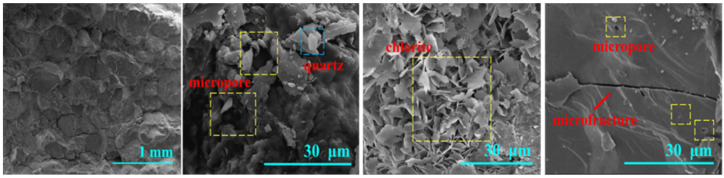
SEM of sandstone sample.

The SEM scanning results of the core are shown in Figure 2.

The results of scanning electron microscopy indicate that the selected cores exhibit a relatively high density and are characterized by a significant presence of particle structures. The structures are mainly filled with flake chlorite and local quartz particles, along with micron-scale pores and microcracks. The aforementioned characteristics are indicative of sandstones with high density and low permeability.

### 3.2. Structure and Performance Analysis of PVE

#### 3.2.1. Enhanced Techniques for Spectral Analysis

**Figure 3:**
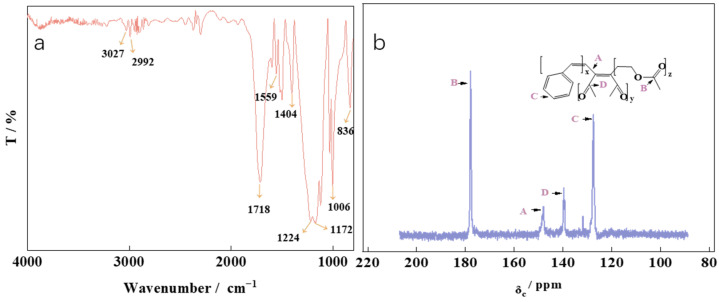
The FT-IR spectra (**a**) and NMR spectra (**b**) of PVE.

The infrared absorption spectrum of the synthetic product is depicted in Figure 3a. The nuclear magnetic spectrum of the product is shown in Figure 3b.

The data in Figure 3a show that the -COOH stretching vibration in VAC exhibits an absorption peak at 1559 cm^−1^, while the ester group C=O stretching vibration in VAC displays an absorption peak at 1718 cm^−1^. The benzene ring carbon skeleton of St demonstrates stretching vibration absorption peaks at 1404 and 836 cm^−1^. Additionally, C-H bonds are observed at 2992 and 3027 cm^−1^, whereas antisymmetric and stretching vibration peaks of C=O in MA are detected at 1224 cm^−1^, 1172 cm^−1^, and 1006.77 cm^−1^. According to the principle of nuclear magnetic carbon spectrum analysis [39,40], the peaks at δ = 127.49 and 131.8 in Figure 3b correspond to benzene ring formations, while the peak at 147.97 represents C=C bonds on VAC. Additionally, the peaks at 139.66 and 139.26 indicate multiple -COO groups in VAC, whereas the peak at 177.95 corresponds to -C-CH_3_ in MA. The combination of FT IR and ^13^C NMR for the product allows it to be identified as a ternary polymer of St-MA-VAc, which is named PVE.

#### 3.2.2. Microstructure Analysis of PVE Using SEM

**Figure 4:**
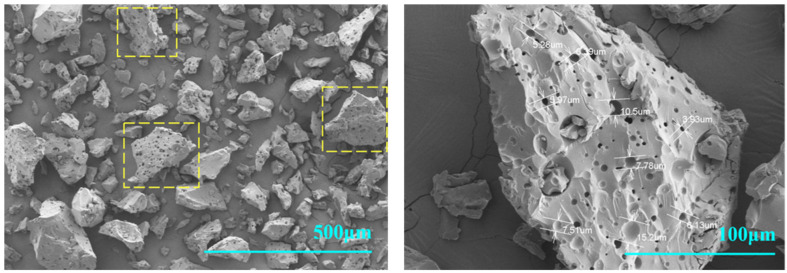
The SEM of the PVE sample powder.

The SEM characterization results of PVE powder are shown in Figure 4.

As shown in Figure 4, the PVE samples are irregularly shaped, massive particles measuring 130 to 190 μm in diameter. The surface of these particles is covered with numerous uniformly distributed pores clearly in the yellow squares, each with a diameter ranging from approximately 5 to 10 μm.

#### 3.2.3. Analysis of Heat Resistance of PVE

**Figure 5:**
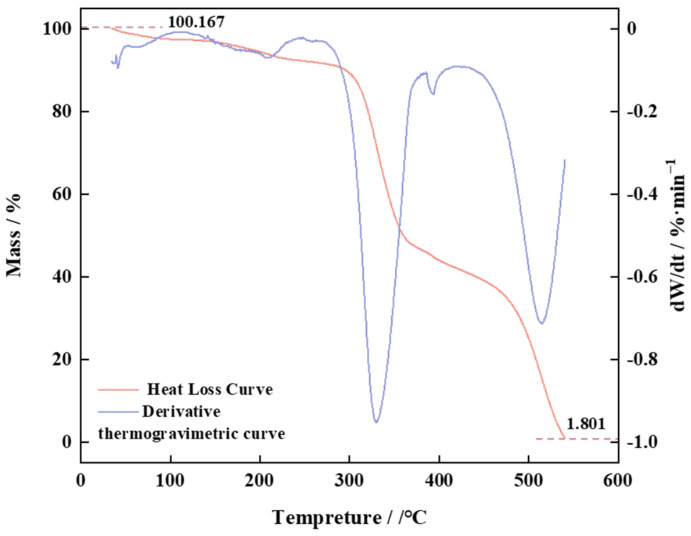
TG and DSC of PVE.

The TG and DSC curves of PVE were investigated experimentally, as shown in Figure 5.

Based on the thermogravimetric and differential curve data of the product, the heat loss process can be classified into four distinct stages. In the first stage, the weight loss at 50–150 °C is measured to be 3.131%, indicating the evaporation of bound water in the sample. During the second stage, the thermal decomposition of the side-chain carboxylic acid in the sample results in a weight loss of 5.525% within the temperature range of 150–280 °C. The third stage occurs at a temperature range of 280–369 °C, during which there is a significant weight reduction caused by the fracture of the main chain (C-C), accounting for 69.4927%. In the fourth stage, at a temperature range of 470–530 °C, the benzene ring undergoes fracture resulting in heat dissipation, leading to a final residue content of 1.801% in the sample that can sustain a stable structure within the temperature range of 80–150 °C. The aforementioned analysis has confirmed the sample. The initial portion of the curve exhibits an excess of 100%, attributed to the preheating and volatilization process of the solvent.

#### 3.2.4. Solubility of PVE in scCO_2_

**Figure 6:**
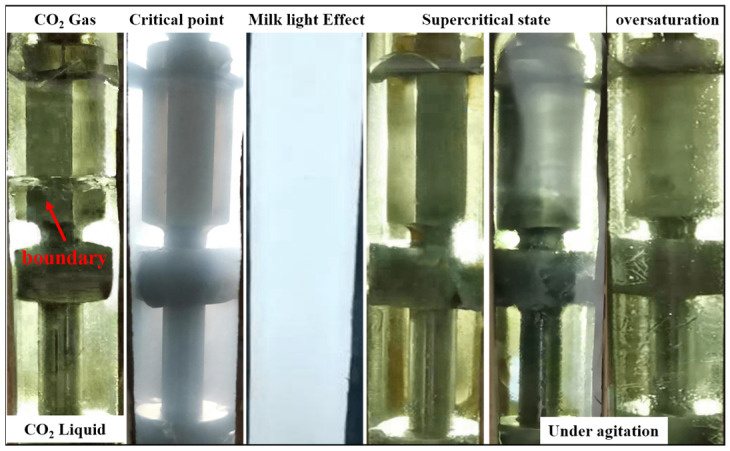
The process of miscibility and dissolution of PVE in scCO_2_.

The observation through the window in Figure 6 revealed that as pressure increased, CO_2_ underwent a gradual liquefaction process, resulting in a progressive rise of the liquid level from the bottom to the top of the window. The light transmittance of the window system decreased as the pressure increased [41], indicating that the system entered the critical phase transition range towards a liquid–supercritical state. After the continuous increase in pressure, the system rapidly reached a state of clarity, indicating that the CO_2_ within the reactor had already transitioned into a supercritical phase. Consequently, further pressure escalation was halted. The recorded reading on the pressure probe at this point corresponded to the minimum miscible pressure (7.80 MPa). The system in the observation window remained uniformly transparent during the stage of low-speed stirring and constant-pressure standing, with no observed presence of insoluble solid substances in the scCO_2_ system. This indicates that PVE, with a mass fraction of 0.1%, can be completely dissolved in scCO_2_ under these environmental conditions. The blank control group reached a supercritical state only when the pressure increased to 7.84 MPa, with a pressure difference of 0.04 MPa between the two, indicating that PVE could decrease the minimum miscible pressure of the system. The decrease in miscible pressure can be attributed to the -COOH group present in the molecular structure of PVE, which enhances the affinity between the thickener molecule and CO_2_, reduces interfacial tension, and ultimately leads to a macro-level reduction in miscible pressure.

The experiment was conducted under constant pressure, and the dosage of thickener was incrementally increased until a small amount of undissolved granular matter was observed in the system; it was concluded that the maximum solubility of the system for PVE at 7.80 MPa was 2.04%. Under a constant temperature of 70 °C, the system pressure was increased to 15 MPa and 20 MPa. It was observed that the saturation solubility of PVE in scCO_2_ exhibited an increase corresponding to the rise in pressure [42], measuring 3.03% and 6.28%, respectively. The solubility of the thickener was also examined at 50 °C, revealing that temperature variations had minimal impact on the supercritical phase transition point of pure carbon dioxide. However, it did lead to a reduction in MMP to 7.77 MPa in a maximum system solubility of 2.20% at this condition. These findings indicate that the saturation solubility of PVE in scCO_2_ decreases with increasing temperature. The reactor speed was adjusted to 3000 revolutions per minute (r/min), and the agitated saturated system was tested under various pressures. The stirring process was influenced by the shear action, leading to the instantaneous gasification of some CO_2_. However, no solute precipitation occurred in any of the groups, and the system could be restored to a uniform and stable state after ceasing the stirring. The influence of shear on the solubility of the system was negligible. When the system pressure exceeded 7.80 MPa, the system exhibited clarity and transparency. However, upon a reduction in pressure below the critical point, a phase transformation turbidity stage occurred, resulting in cloudiness within the system. Nevertheless, the system was still homogeneous, with no solid material precipitation, proving that the sample can be soluble in supercritical carbon dioxide. The current test results demonstrate that the solubility of PVE in scCO_2_ remains significantly high, which can be attributed to the robust internal thermal stability of the product below 150 °C and the enhanced molecular thermal motion facilitating dissolution in a high-temperature environment. Rapid dissolution is facilitated by the porous structure, which effectively reduces surface tension.

### 3.3. The Impact Assessment of PVE

#### 3.3.1. Viscosification Effect Evaluation

**Figure 7:**
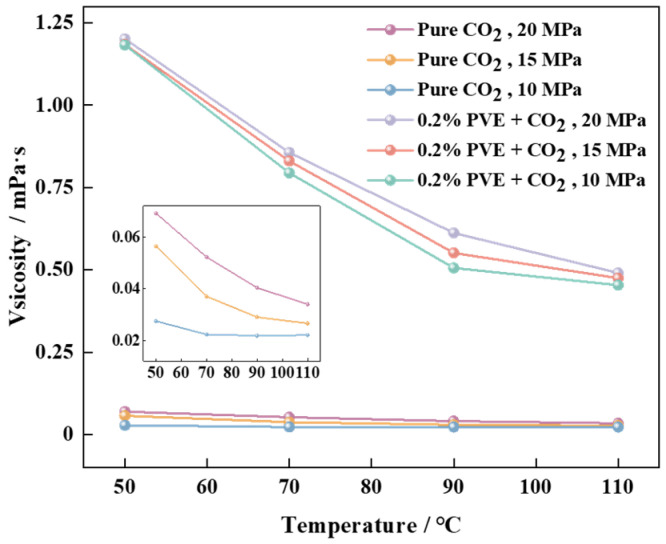
The temperature-dependent shear viscosity curve of the system under various pressures.

The thickening property of the system was evaluated using the rotational volume method [42]. The measurement of torque resistance was conducted under preset conditions of ambient pressure, temperature, and shear velocity. It should be noted that a higher level of resistance indicates an increased viscosity. By considering the rationality of oil displacement cost and the pressure range of the instrument, a mass concentration of 0.2% was selected as the test amount, and the corresponding test results are depicted in Figure 7 [43,44].

The viscosity of the scCO_2_ system and pure scCO_2_ system containing PVE decreases with increasing temperature, as illustrated in Figure 7. Conversely, the viscosity of the system increases with increasing pressure. The addition of 0.2% thickener PVE effectively reduces the viscosity of scCO_2_, as observed when compared to the viscosity of pure scCO_2_ under identical environmental conditions. The shear viscosity of the scCO_2_ system with 0.2% PVE at 10 MPa and 50 °C is measured to be 1.1831 mPa·s, which exhibits a significant increase of 43.04 times compared to pure scCO_2_ under identical conditions. The system is capable of sustaining a viscosity increase of 19.53 times at 10 MPa and 110 °C. The shear viscosity of the scCO_2_ system with 0.2% PVE is measured to be 1.2018 mPa·s (which is equivalent to 16.3876 times 0.0691) at 20 MPa and 50 °C. The viscosity of the system increases by a factor of 13.43 at 20 MPa and 110 °C. The viscosity retention rate of the PVE-scCO_2_ system is 40.06% when the temperature increases from 50 to 110 °C under a constant pressure of 15 MPa. At 70 °C, the viscosity of the PVE-scCO_2_ system reaches 0.8305 mPa·s and undergoes a thickening effect by a factor of 21.43. The van der Waals force between the CO_2_-philic groups C=O and -COOH on the molecules of thickener PVE and CO_2_ in the system is significantly stronger compared to that between CO_2_ molecules, resulting in a deceleration of molecular movement rate and an enhancement of system viscosity [45,46]. The increase in temperature leads to elevated molecular collision and intramolecular energy, resulting in a decrease in van der Waals force and intermolecular spacing. This promotes enhanced intermolecular movement, ultimately reducing viscosity and enhancing system fluidity. The viscosity of the system is minimally affected by low pressure, but as pressure increases, the reduction in free volume between molecules leads to an increase in intermolecular friction and, subsequently, an increase in viscosity [47].

#### 3.3.2. Effect of PVE on Enhancing scCO_2_ Flooding and Recovery

**Figure 8:**
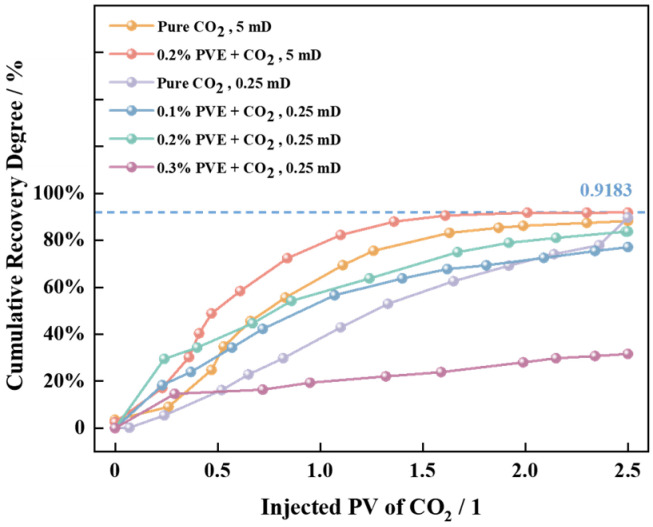
The curve of experimental results for scCO_2_ injection displacement.

The test environment was set to 15 MPa and 70 °C, according to the actual conditions of the target oilfield, for investigating the impact of thickener PVE on the scCO_2_ flooding effect, as depicted in Figure 8.

The results obtained from the permeability cores with 0.25 mD and 5 mD demonstrate that the addition of a 0.2% thickener to the 5 mD core enables an effective extraction of 2.65%. The results indicate that the incorporation of PVE thickener with a mass concentration ranging from 0.1 to 0.2% can further enhance the degree of recovery, and there exists a direct promotion between the degree of recovery and the concentration of introduced thickener. The recovery rate of PVE-scCO_2_ flooding, which is 0.2%, exhibits a significant increase of 5.61% compared to that achieved by pure scCO_2_ flooding. The oil displacement effect deteriorates when the thickener concentration reaches 0.3% due to excessively high system viscosity and reduced migration velocity in the pore throat, thereby impeding the sweep effect. This indicates that the thickener has a specific application range (0.1~0.2%) for low-permeability core displacement.

#### 3.3.3. Analysis of Alterations in Core Permeability and Crude Oil Composition

**Figure 9:**
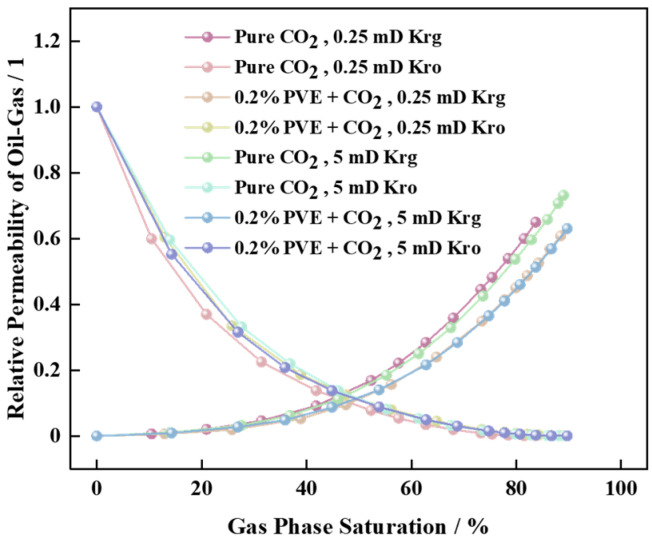
Variation trend of Kro and Krg curves before and after the introduction of PVE.

To gain a deeper understanding of the alterations in pore structure and crude oil composition before and after displacement, the phase permeability curves of pure scCO_2_ as well as 0.2% PVE-scCO_2_ core- and system-produced liquid were determined and subjected to quantitative analysis using gas chromatography. The resulting phase permeability change curves are depicted in Figure 9, and the component data are shown in Table 2.

The change law of the phase permeability curve indicates that the introduction of a thickening agent reduces gas phase permeability (Krg) and increases oil phase permeability (Kro). Consequently, thickened supercritical carbon dioxide flooding effectively inhibits gas channeling and promotes improved oil displacement efficiency.

According to the component analysis in Table 2, both scCO_2_ flooding and PVE-scCO_2_ contribute to the extraction of light components in crude oil, while the polymer itself has minimal impact on component extraction. As the permeation channel of a 5 mD core is more pronounced than that of a 0.25 mD core, the gas flow rate increases and contact time between carbon dioxide and crude oil decreases, resulting in a gradual reduction in the displacement effect on asphaltene and non-hydrocarbon components with increasing permeability. The total amount in the table is less than 100% due to the presence of other substances, such as mineral particles and heteroatoms, in certain extracted portions. However, these substances do not impact the overall composition assessment.

### 3.4. Analysis of Molecular Dynamics (MD) Calculation Results

**Figure 10:**
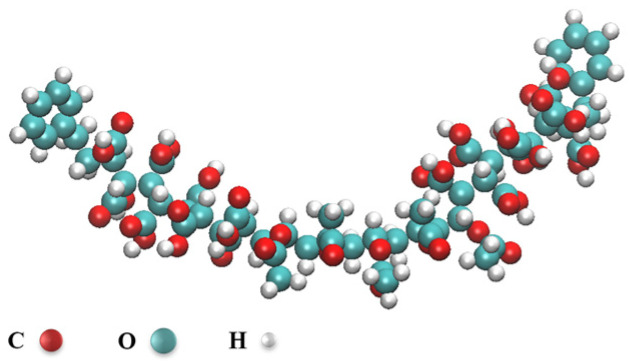
PVE molecule.

The symmetric structure is considered to possess the highest thermal stability among the various isomers that can occur in polymers, based on empirical evidence. To facilitate the discussion, this paper adopts a symmetrical polymer structure where St is directly connected to MA at both ends of the molecule, and VAC is positioned in the center of the linear polymer, as illustrated in Figure 10. The molecule consists of a total of 190 atoms, with 68 being carbon (C) atoms, 80 being hydrogen (H) atoms, and 42 being oxygen (O) atoms. The dimensions of the molecule are measured as 35.766 A × 19.86 A × 9.736 A.

**Figure 11:**
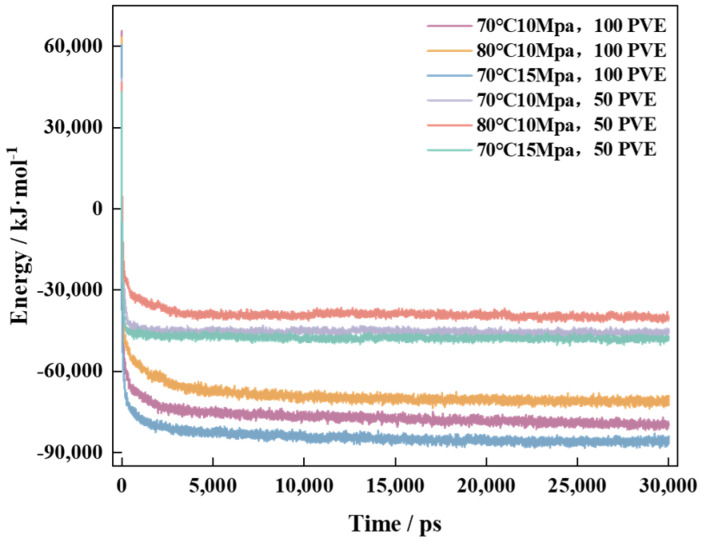
Change in energy balance in PVE-scCO_2_ system.

To simulate real field application conditions, in this study, a 10 × 10 × 20 nm^3^ box containing varying quantities (50/100) of polymer PVE and 4000 CO_2_ was designed. The relationship between area and viscosity was examined at different temperatures and pressures. The systems with 50 PVE-4000 CO_2_ and 100 PVE-4000 CO_2_ were investigated at 70 °C and 10 MPa (343.25 K and 100 bar), 80 °C and −10 MPa (353.25 K and 100 bar), and 70 °C and −15 MPa (343.25 K and 150 bar). The MD simulation process initially underwent energy minimization, followed by a 5 ns NVT ensemble simulation and subsequently a 30 ns NPT ensemble simulation to investigate the attainment of the total energy balance within the system [49] (Figure 11).

**Figure 12:**
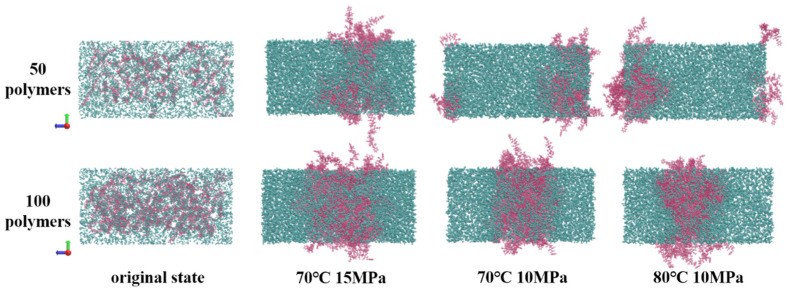
The molecular distribution in each system before and after reaching equilibrium.

According to the analysis of the energy curve’s movement law, during the NPT simulation process, there is a rapid decrease in system energy within the first 5 ns of the simulation. As it reaches 5 ns, each system’s energy tends to reach equilibrium. The energy convergence rate of the system is faster at 70 °C and −15 MPa compared to 70 °C and −10 MPa, while it is slower at 80 °C and −10 MPa. Furthermore, as the temperature decreases and pressure increases, the system exhibits a higher tendency to converge toward its equilibrium state. The increase in temperature enhances the thermal motion of molecules, which hinders the system’s equilibrium, whereas pressure has the opposite effect [50]. The systems converge to different energy values under the same conditions due to variations in the amount of polymer introduced, with a greater number of polymer molecules resulting in higher energy loss during final convergence to equilibrium (Figure 12). After the system’s equilibrium was confirmed, the distribution of polymers in the system was examined using the visualization software VMD _win64-1.9.3. The “.gro” file of the system was color-coded in the interface to facilitate differentiation, with PVE molecules labeled as PINK and CO_2_ molecules labeled as ICEBLUE due to their abundance of C and O elements.

**Figure 13:**
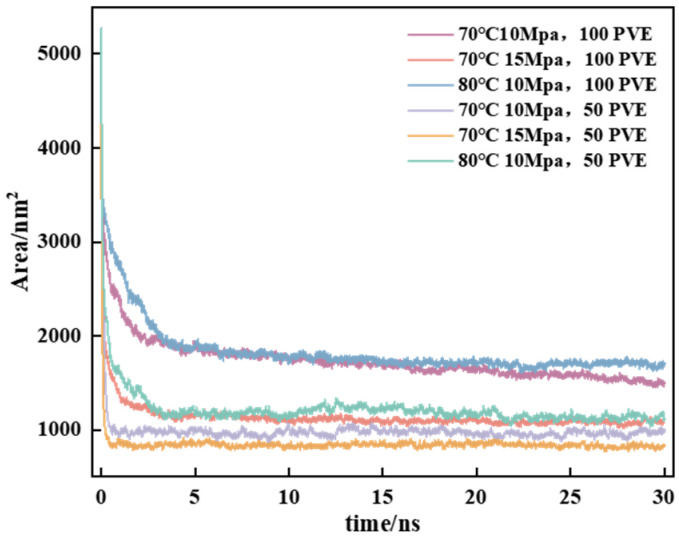
The total contact area of the CO_2_-PVE system in each individual system under various conditions.

The idealized design of the initial state in Figure 12 only considers a uniform distance distribution, neglecting the full consideration of intermolecular forces. Consequently, the polymer and carbon dioxide exhibit an unrealistic state within the box. After equilibrium is reached, the molecular distribution of polymer molecules undergoes changes due to the influence of van der Waals forces, leading to a gradual transition towards an aggregated state. To gain a deeper understanding of the interaction between aggregates and carbon dioxide, the total contact area of the system was computed, and the resulting changes in area are summarized in Figure 13.

**Figure 14:**
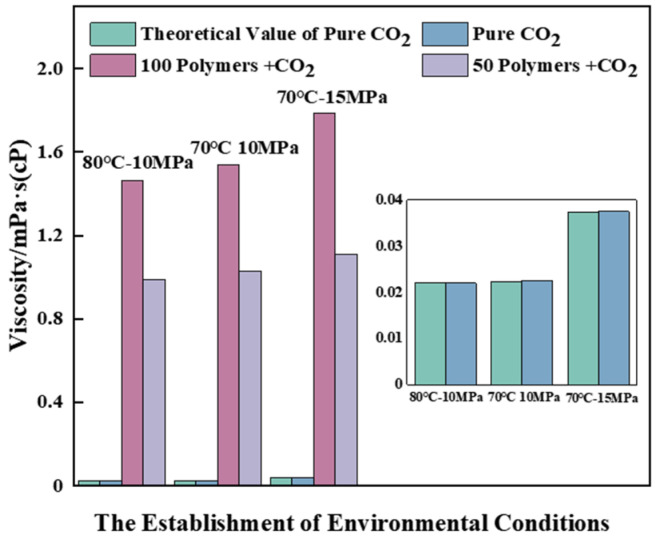
The viscosity changes of each system compared under various conditions.

According to the law of curve change, it can be observed that the transition from an imbalanced uniform state to an equilibrium state results in a decrease in the total contact area of the medium system. The results demonstrate that PVE enhances the overall contact area of PVE-CO_2_ under high-temperature and low-pressure conditions. The total contact area of the system exhibited a positive correlation with the number of polymer molecules. The 2 ps finished product simulation was continued using the periodic disturbance method, in order to investigate the viscosity changes of the system depicted in Figure 14, while maintaining its dynamic equilibrium.

The calculated data of pure carbon dioxide MD under basic conditions (70 °C, 10 MPa) were compared with the theoretical standard value [51]. The resulting calculated viscosity was found to be 0.02250 mPa·s. This value was then compared to the theoretical standard viscosity of 0.0235 mPa·s, revealing an error rate of only 0.043%. These findings demonstrate the high reliability of our calculations. The Green–Kubo viscosity formula was employed for the calculation of viscosity [52]:

(1) $$\eta =\frac{V}{k_{B}T}{\int_{0}^{\infty }\langle P_{xz}\left(t_{0}\right)P_{xz}\left(t_{0}+t\right)\rangle }_{t_{0}}dt$$

where variables k_B_, *V*, and *T* represent the Boltzmann constant, temperature, and volume of the simulated box, respectively.

After an analysis of the viscosity data in Figure 14, it can be inferred that the polymer exhibits a significant increase in viscosity, indicating its effectiveness. The impact of pressure on viscosity is significantly more pronounced than that of temperature under identical component conditions. The viscosity of each system exhibits the most significant increase at 10 MPa and 50 °C, with 100 PVE-4000 CO_2_ showing a remarkable enhancement of 68.44 times and 50 PVE-4000 CO_2_ demonstrating an impressive amplification of 45.81 times. Notably, these changes align precisely with the experimental observations. The increase in the number of introduced polymers can be deemed to have a positive impact on the viscosity alteration of the system, aligning with the aforementioned rule regarding changes in contact area. Therefore, expanding the effective contact area is a viable approach to enhance the viscosity of scCO_2_.

## 4. Conclusions

The polymer PVE was synthesized in this study, and the solubility test demonstrated its favorable affinity towards CO_2_. At a temperature of 70 °C, PVE with a mass fraction of 0.1% can be dissolved in scCO_2_, resulting in a significant reduction in the minimum miscible pressure to 7.84 MPa for the system. At this stage, the saturation solubility of the polymer in scCO_2_ is predominantly influenced by variations in pressure rather than shear. The viscosity of the scCO_2_ system can be effectively increased by 15 MPa within the concentration range of 0.1–0.3%. Additionally, at a temperature of 70 °C, the addition of 0.2% P results in a remarkable increase in shear viscosity, reaching a value that is 21.43 times higher than that without PVE. The thickener concentration range for a 0.25 mD core is 0.1–0.2%. In this scenario, the recovery efficiency of the displacement system increases with higher thickener concentrations. The recovery efficiency of 0.2% P-scCO_2_ flooding is 5.61% greater than that of pure scCO_2_ flooding. The introduction of a thickener is believed to effectively suppress gas channeling and enhance the extraction of light components during scCO_2_ flooding, as evidenced by the permeability curve of the core phase and changes in crude oil composition before and after thickening. Moreover, it exerts minimal influence on asphaltene deposition. Molecular simulation analysis reveals that the viscosity error rate with the theoretical standard is merely 0.043%, indicating high calculation reliability. The analysis of the simulation rule indicates a positive correlation between polymer viscosity and both the contact area of carbon dioxide and the number of polymers. Moreover, the observed change in viscosity is consistent with experimental determinations.

This study not only presents a novel and effective approach for designing high-performance CO_2_ thickeners in high-temperature and high-pressure environments, but also elucidates the molecular-level viscosification mechanism of polymer-SCCO_2_ systems. It identifies crucial areas for future research on scCO_2_ viscosification technology and provides a fresh perspective for developing efficient oil displacement systems.

## Figures and Tables

**Table 1:** The pros and cons of various gas flooding applications.

Gas Type	Specific Mechanism	Advantages	Limitations
CO_2_	Extraction and gasification of light hydrocarbons to form mixed phases.	High compression ratio and excellent miscibility with crude oil [7]. Contributes to reducing greenhouse gas emissions and pollution.	Gravity differentiation leads to gravity overlap phenomenon. Restricted injection gas source. Strong corrosivity [8]. Asphalt precipitation due to light hydrocarbon extraction effects.
N_2_	It is capable of extracting light hydrocarbons, expanding the heating zone of oil reservoirs, and enhancing the efficiency of oil displacement and profile control.	Abundant resources, competitive pricing, negligible corrosion concerns, robust suction capacity, easy attainment of injection–production equilibrium, minimal gas channeling issues, higher compression coefficient compared to gas cap gases, and larger quantities of flue gas and CO_2_. These factors contribute to enhanced formation energy while being minimally affected by salinity.	The miscibility condition surpasses that of CO_2_ and hydrocarbon gas, necessitating higher injection pressure and resulting in a narrower application range.
Flue Gas	Combination of carbon dioxide and nitrogen displacement mechanism.	Enhance environmental sustainability and economic viability by mitigating greenhouse gas emissions and reducing pollution.	Insufficient air supply and high transportation costs. Improper treatment can result in water contamination in the gas, leading to pipeline and equipment corrosion. The initial investment is substantial, with slow returns and high risks. For reservoirs with high permeability, gas flow is facilitated, but effectiveness may be limited.
Hydrocarbon Gas	The evaporation and extraction miscible effects are limited [9].	The pressure of miscibility is low, resulting in a suboptimal outcome.	The air supply is constrained, and the cost is comparatively elevated.
Air	The low-temperature oxidation of crude oil consumes oxygen and generates heat, resulting in a gravity-driven displacement at the top of thick or inclined reservoirs.	The source is extensive, unrestricted by geographical boundaries, abundant in air resources, and cost-effective.	There are limits on reservoir thickness [10], and oxygen corrosion takes place [11].

**Table 2:** The percentage composition of crude oil components ^1^ (%).

Source of Oil Samples	Alkane, wt%	Aromatic, wt%	Non-Hydrocarbon, wt%	Asphaltene, wt%	Gross, wt%
Crude Oil	67.52	11.58	8.04	2.89	90.03
0.25 mD	70.01	11.62	8.05	2.58	92.26
0.25 mD + PVE	69.83	11.63	8.03	2.67	92.16
5 mD	68.52	11.59	8.01	2.88	91
5 mD + PVE	68.43	11.61	8.03	2.88	90.96

^1^ The data in the table have been calculated using the “area percentage method” [48].

## Data Availability

Data are contained within the article.
